# Insights Into the Pathogenesis of Sweet's Syndrome

**DOI:** 10.3389/fimmu.2019.00414

**Published:** 2019-03-12

**Authors:** Michael S. Heath, Alex G. Ortega-Loayza

**Affiliations:** Oregon Health and Science University, Department of Dermatology, Portland, OR, United States

**Keywords:** acute febrile neutrophilic dermatosis, neutrophilic dermatoses, malignancy, drug induced, autoinflammation, clonality, hematology

## Abstract

Sweet's syndrome, also known as Acute Febrile Neutrophilic Dermatosis, is a rare inflammatory condition. It is considered to be the prototype disease of neutrophilic dermatoses, and presents with acute onset dermal neutrophilic lesions, leukocytosis, and pyrexia. Several variants have been described both clinically and histopathologically. Classifications include *classic Sweet's syndrome, malignancy associated, and drug induced*. The cellular and molecular mechanisms involved in Sweet's syndrome have been difficult to elucidate due to the large variety of conditions leading to a common clinical presentation. The exact pathogenesis of Sweet's syndrome is unclear; however, new discoveries have shed light on the role of inflammatory signaling, disease induction, and relationship with malignancy. These findings include an improved understanding of inflammasome activation, malignant transformation into dermal infiltrating neutrophils, and genetic contributions. Continued investigations into effective treatments and targeted therapy will benefit patients and improve our molecular understanding of inflammatory diseases, including Sweet's syndrome.

## Introduction

Sweet's syndrome (SS) was originally described as “acute febrile neutrophilic dermatosis” by Sweet, (1). His original report was based on the clinical-pathologic presentation of 8 women who presented with acute onset fever, leukocytosis and erythematous, tender plaques with dense neutrophilic infiltration in the dermis. These patients had no evidence of infection and had rapid response to systemic corticosteroids. As additional reports of this newly described pathologic entity surfaced, the syndrome was renamed to recognize Dr. Sweet (2). Subsequent to these initial accounts, thousands of cases have been described in literature. This led to a better understanding and recognition of a multitude of clinical variants and SS classifications. Unfortunately, due to the rarity of SS, epidemiologic information including incidence is unknown.

The traditional description of tender erythematous plaques and nodules remains the prototypical presentation. However, clinical variants including localized neutrophilic dermatosis of the dorsal hands, bullous, subcutaneous, cellulitic, and necrotizing lesions have been reported (3–7). Extracutaneous manifestations have also been reported including involvement with the central nervous system, internal organs and musculoskeletal system (8–10). Histopathologic variants include histiocytoid SS and SS with vasculitis which has been hypothesized to be a secondary reaction (11, 12).

SS is one pathologic entity within the broader neutrophilic dermatoses classification. Neutrophilic dermatoses include SS, pyoderma gangrenosum, neutrophilic eccrine hidradenitis, and Behçet's disease among others. Each disease has some overlapping pathophysiology with an autoinflammatory component made up of predominately neutrophilic infiltrate. Each entity is distinguished by disease chronicity, tissue involvement, and clinical appearance. Understanding the pathogenesis of SS is important from a diagnostic and therapeutic perspective. In a time of revolution in immunology and targeted therapy the pathways discovered in SS can have broader implications in additional autoinflammatory diseases as well as malignancy.

## Disease Classifications and Associations

SS has been associated with a multitude of diseases, malignancies and medications at varying frequencies (Table 1). Given the unpredictable nature of the disease, it has been difficult to reach conclusions regarding true associations and causations. The temporal relationships and frequency of concurrent processes has led to the recognition of several pathologic relationships. Some authors agree that there are three distinct variants which are important to distinguish, given differential work up and management recommendations. These three subtypes are Classic SS, Malignancy Associated SS, and Drug Induced SS and will be discussed individually and are summarized in Table 1.

**Table 1:** Conditions and medications coexisting in Sweet's Syndrome in descending order of referenced literature.

**Classic SS**				**Malignancy associated SS**		**Drug induced SS**	
**Autoimmune and autoinflammatory conditions**		**Infectious etiologies**		**Hematologic malignancies**			
Ulcerative Colitis	(13–29)	NTM	(30–42)	AML	(43–68)	G-CSF or GM-CSF	(55, 69–87)
Crohn's Disease	(66, 88–104)	HIV	(105–111)	MDS	(43, 112–134)	Azathioprine	(135–147)
Erythema nodosum	(128, 148–159)	TB	(160–166)	CML	(43, 44, 167–177)	ATRA^*^	(54, 178–188)
Sarcoidosis	(152, 154, 189–196)	URI	(197–200)	APL^*^	(178–187, 201)	Hydralazine	(202–207)
SLE	(208–214)	Hepatitis C Virus	(110, 194, 215, 216)	Multiple myeloma	(217–226)	Bortezomib	(218, 227–231)
Relapsing Polychondritis	(117, 119, 120, 124, 232–236)	Gastroenteritis	(237–241)	Hairy Cell leukemia	(37, 242–249)	TMP-SMX	(250–254)
Vasculitis	(114, 255–262)	Varicella Zoster Virus	(211, 263)	CLL	(264–269)	Tetracyclines	(270–274)
PG	(17, 28, 275, 276)	Cytomegalovirus	(277, 278)	Hodgkin's lymphoma	(279–281)	NSAID	(282–286)
MSO	(9, 287–289)	Hepatitis B Virus	(290, 291)	Non-Hodgkin's lymphoma	(292, 293)	Azacitidine	(294–299)
Behcet's disease	(300–305)	Parvovirus B19	(306, 307)	CNL	(308)	Vaccination	(108, 309–313)
Ankylosing spondylitis	(15, 91, 314)	Chlamydial Infection	(315, 316)	ALL	(46)	Oral Contraceptive	(317–319)
Rheumatoid arthritis	(164, 320–323)	Herpes Simplex Virus	(36, 324–326)	Juvenile MML	(327)	Lenalidomide	(328, 329)
SCLE	(330–332)	Bacterial Endocarditis	(333–335)	Juvenile CML	(336)	Ipilimumab	(337–340)
Subacute thyroiditis	(341)	Cellulitis	(342–344)	EATL	(345)	Imatinib	(170, 346, 347)
Hashimoto's thyroiditis	(348–350)	Capnocytophaga	(351)	DLBCL	(352)	Vemurafenib	(353, 354)
Autoimmune hepatitis	(278, 355)	Biliary sepsis	(356)	DHL	(357)	Furosemide	(358)
Bronchiolitis obliterans	(359, 360)	Dermatophyte	(361)	CTCL	(362)	Adalimumab	(15, 363)
Cryptogenic pneumonia	(364, 365)	Francisella tularensis	(366)	B cell lymphoma	(367)	Interferon β – 1b	(368, 369)
Multiple sclerosis	(368, 370)	Glandular Tularemia	(371)	**Solid Tumor Malignancies**		Isotretinoin	(372, 373)
Sjogren's syndrome	(164, 374)	Helicobacter pylori	(375)	Breast carcinoma	(268, 324, 376–380)	Sulfasalazine	(381, 382)
Unknown arthritis	(383–385)	HG anaplasmosis	(386)	Prostate Cancer	(133, 387–391)	Clindamycin	(392, 393)
Aseptic meningitis	(394)	Klebsiella cystitis	(395)	Oral SCC	(396–399)	Clozapine	(400, 401)
Autoimmune cholangitis	(265)	Pasteurella multocida	(402)	Cervical cancer	(165, 403, 404)	IL-2 therapy	(405)
Celiac disease	(406)	PCP	(57)	Gastric cancer	(407–410)	Abacavir	(411)
Cryptogenic cirrhosis	(275)	Coccidioidomycosis	(412)	Lung cancer	(217, 413–415)	APAP-codeine	(416)
Dermatomyositis	(417)	Salmonella typhimurium	(418)	Melanoma	(337–339)	Allopurinol	(419)
Dressler's Syndrome	(420)	Sporotrichosis	(421)	Ovarian carcinoma	(422, 423)	Dabrafenib/trametinib	(424)
FMF	(425)	**Other**		Testicular cancer	(426, 427)	Carbamazepine	(428)
Granuloma annulare	(377)	Pregnancy	(19, 429–438)	Bladder Cancer	(389, 439)	Decitabine	(440)
Grave's Disease	(441)	Trauma	(416, 442–450)	Thyroid Carcinoma	(451)	Diazepam	(452)
Hypothyroidism	(453)	Radiation therapy	(177, 398, 454–459)	Adrenal cortex carcinoma	(460)	Fluconazole	(461)
IHCP	(462)	Photoinduced	(463–465)	Merkel cell carcinoma	(466)	Gabapentin	(467)
Myasthenia gravis	(468)	Chronic Lymphedema	(469–474)	Osteosarcoma	(475)	Infliximab	(476)
Pigmented villonodular synovitis	(477)	Fanconi Anemia	(478–481)	Pheochromocytoma	(482)	Ketoconazole	(483)
Pemphigus vulgaris	(484)	Polycythemia Vera	(485–489)	Tonsil cancer	(490)	Mesalamine	(491)
Still's disease	(492)	Myelofibrosis	(167, 459, 468, 493–497)	Liposarcoma	(498)	Hormonal IUD	(499)
Subacute necrotizing lymphadenitis	(500)	Other Immunodeficiency	(31, 501–503)	Gallbladder adenocarcinoma	(504)	Mitoxantrone	(370)
SAPHO	(505, 506)			Esophageal Adenocarcinoma	(507)	Nitrofurantoin	(508)
Autoimmune thyroiditis	(509)			Rectal adenocarcinoma	(510)	Norfloxacin	(388)
Connective tissue disorder	(511)					Ofloxacin	(90)
						Piperacillin and tazobactam	(265)
						Propylthiouracil	(512)
						Proton pump inhibitor	(378)
						Quinupristin and dalfopristin	(513)
						Ruxolitinib	(494)
						Ticagrelor	(514)
						Topotecan	(515)
						Vedolizumab	(516)
						Vorinostat	(297)

* *High proportion of reported Sweet's syndrome cases associated with acute promyelocytic leukemia also received ATRA*.

*ALL, Acute lymphoblastic leukemia; AML, Acute myeloid leukemia; APAP, Acetaminophen; APL, Acute promyelocytic leukemia; ATRA, All-trans retinoic acid; CGD, Chronic granulomatous Disease; CLL, Chronic lymphocytic leukemia; CML, Chronic myelogenous leukemia; CNL, Chronic neutrophilic leukemia; CTCL, Cutaneous T-cell lymphoma; CVID, Common Variable Immunodeficiency; DLBCL, Diffuse large B-cell lymphoma; DHL, Diffuse histiocytic lymphoma; EATL, Enteropathy-associated T cell lymphoma; FMF, Familial Mediterranean Fever; G-CSF, Granulocyte-colony stimulating factor; GM-CSF, Granulocyte macrophage-colony stimulating factor; HIV, Human immunodeficiency virus; HG, Human granulocytic; IHCP, Idiopathic hypertrophic cranial pachymeningitis; IL-2, Interleukin-2; IUD, Intrauterine device; MDS, Myelodysplastic syndrome; MML, myelomonocytic leukemia; MRSA, Methicillin-resistant Staphylococcus aureus; MSO, Multifocal sterile osteomyelitis; NSAIDs, Non-steroidal antiinflammatory drug; NTM, Non-tuberculous mycobacterium; PCP, Pneumocystis carinii; PG, Pyoderma Gangrenosum; SAPHO, Synovitis, acne, pustulosis, hyperostosis, and osteitis; SCC, Squamous cell carcinoma; SCLE, Subacute cutaneous lupus erythematosus; SLE, Systemic Lupus Erythematosus; TB, Tuberculosis; URI, Upper respiratory infection*.

### Classic Sweet's Syndrome (Idiopathic Sweet's Syndrome)

Classic SS is responsible for most SS cases and has a predilection for women. Initial presentation most frequently occurs between age 30 and 60 years (517), but has been reported in multiple pediatric patients including neonates in the first 10 days of life (518). Although considered idiopathic, it has been reported in association with infections, pregnancy, and inflammatory and autoimmune disorders among others (Table 1) (13, 30, 330, 435).

Diagnostic criteria for classic SS was proposed by Su and Liu and updated by von den Driesch (254, 519). Diagnosis is based on fulfilling both major criteria and two of the four minor criteria which are presented in Table 2.

**Table 2:** Diagnostic Criteria for Classic Sweet's Syndrome.

**MAJOR CRITERIA**
Abrupt onset of painful erythematous plaques or nodules Histopathologic evidence of a dense neutrophilic infiltrate without evidence of leukocytoclastic vasculitis
**MINOR CRITERIA**
Fever >38°C Associated with inflammatory disease or pregnancy or preceded by upper respiratory infection, gastrointestinal infection, or vaccination Excellent response to treatment with systemic glucocorticoids or potassium iodide Abnormal laboratory values at presentation (three of four of the following): Erythrocyte sedimentation rate >20 mm/h Positive C-reactive protein >8,000 leukocytes per microliter >70% neutrophils

### Drug Induced Sweet's Syndrome

The most commonly reported drug associations are Granulocyte-colony stimulating factor (G-CSF), Azathioprine, and All-trans retinoic acid (ATRA). Most other etiologies are infrequent (Table 1). Diagnostic criteria for drug induced SS was suggested by Walker and Cohen (250). It requires all five criteria summarized in Table 3 be met to establish the diagnosis.

**Table 3:** Diagnostic Criteria for Drug Induced Sweet's Syndrome.

Abrupt onset of painful erythematous plaques or nodules Histopathologic evidence of a dense neutrophilic infiltrate without evidence of leukocytoclastic vasculitis Fever >38° Temporal relationship between drug ingestion and clinical presentation, or temporally-related recurrence after oral challenge Temporally-related resolution of lesions after drug withdrawal or treatment with systemic corticosteroids

### Malignancy-Associated Sweet's Syndrome

It has been suggested that the first reported case of malignancy associated SS was published by Costello 9 years prior to Sweet's disease defining paper (520). Malignancy, both solid tumor and hematologic, have been reported in a large proportion of SS cases (Table 1) (521). Specific SS characteristics may represent an increased risk of malignancy, including subcutaneous and histiocytoid histopathologic variants (522, 523). Diagnostic criteria for malignancy associated SS is the same as classic SS, except for the substitution of “an underlying malignancy” as a minor criterion rather than “an inflammatory disease, pregnancy, vaccination or infection” (254, 519).

## Pathogenesis

### Neutrophil Proliferation and Maturation

Just as the associated condition and etiology of SS varies considerably, the pathogenesis is multifactorial and likely non-uniform between subtypes of the disease. The inciting activator of SS, especially classic SS, has not been determined, although cases of hematologic malignancy and initiation of granulocyte colony stimulating factors (G-CSF), all-trans retinoic acid (ATRA), and fms-like tyrosine kinase-3 (FLT3) inhibitors offer a glimpse into one mechanism. G-CSF acts within the bone marrow, serum and tissue, causing neutrophil differentiation, maturation and activation. As a response to pathogens, G-CSF is a part of the innate immune system signaling which is maladaptively elevated in inflammatory states (524). In cases of classic SS, patients with an underlying infection or autoimmunity, the pathologic increase in colony stimulating factors may be the causative agent (525, 526). Endogenously elevated G-CSF levels have been reported in multiple cases of SS, with elevations in serum concentrations correlating with clinical disease severity (127, 524). *In vitro*, SS neutrophils have high rates of apoptosis when isolated. Conversely, when cultured with serum from SS patients, the apoptosis rate is significantly decreased and neutrophil survival is significantly greater (524). This serum enhanced survival suggests elevated G-CSF among other circulating factors contribute to the disease. Both solid tumor and hematologic malignancies can produce colony stimulating factors. In malignancy-associated SS, this paraneoplastic phenomenon might represent an inciting factor in disease progression (127, 527–529). The frequency of drug-induced SS from the exogenous use of G-CSF further reinforces the causative role of G-CSF in SS (517, 530–533). After initiation of G-CSF therapy in SS associated with hematologic malignancies, it is theorized that G-CSF induces differentiation and maturation of leukemic cells which then home to the skin (55, 534). Similarly, ATRA induces the differentiation of promyelocytes in acute promyelocytic leukemia (APL). ATRA has been associated with developing SS in APL and the mature dermal neutrophils may be progeny from differentiated malignant cells. This is evidenced by sequential SS lesional biopsies showing gradual maturation of neutrophils in the dermis mirroring neutrophil maturation in the peripheral blood (181).

### Malignant Transformation

Investigations have shown neutrophilic clonality within SS lesions suggestive of either hematologic malignancy transformation into mature dermal neutrophils or localized non-malignant neutrophil stemming from a common dysfunctional progenitor (535, 536). Analysis with fluorescent *in situ* hybridization have shown the SS lesional neutrophils exhibit the same genetic abnormalities as the underlying malignant myeloblasts in serum and bone marrow, suggesting a clonal transformation into dysplastic neutrophils in the dermis (49, 55, 534, 537, 538). Recently, examination of the bone marrow and SS lesional tissue in a patient with concurrent acute myeloid leukemia (AML) with single nucleotide polymorphism array and next generation sequencing revealed FLT-3 gene mutations in infiltrating mature neutrophils and neoplastic progenitor cells (539). In one case series, FLT-3 mutations have been detected in 39% of patients with AML and SS and FLT-3 inhibitors are a known SS inducer (49, 540, 541). This gene encodes a receptor tyrosine kinase normally present on hematopoietic stem cells within the bone marrow and regulates myeloid progenitor cell proliferation, survival, and differentiation (542). In AML the FLT-3 mutations result in persistent activation. The identification of this mutation in dermal neutrophils and leukemic cells suggests a common progenitor origin.

### Induction and Stimulus

Given the variety of underlying conditions including medications, infections, and malignancy associated with a similar clinicopathologic presentation in SS, one unifying hypothesis is that SS is a hypersensitivity reaction. Immune reaction to drugs, bacterial, viral, or tumor antigens may initiate a cytokine cascade resulting in SS (3). The efficacy of systemic corticosteroids and resolution of SS with treatment of underlying disease with antibiotics or chemotherapy supports this hypothesis, but there is a lack of evidence showing immune-complexes, immunoglobulins or changes in complement consistent with a hypersensitivity reaction (11, 519, 543).

Photoinduction and Koebner phenomenon have also been suggested as possible inciting etiologies in SS and may explain the distribution and localization to the skin (544). Photoinduction of SS has been documented and confirmed in select patients with experimental phototesting re-challenge (464, 545–549). While not fully elucidated, a proposed mechanism is founded on the immunomodulating effects of light. The most notable concept involves the pro-inflammatory potential of ultraviolet B in activating neutrophils and inducing the production of TNF-α and interleukin-8 (548, 550, 551). The formation of SS lesions in response to localized trauma has been demonstrated by lesions developing at sites of radiation therapy, surgery, burns, tattoos, and lymphedema (442–445, 454–457, 472, 474).

### Cutaneous Localization

Localization of neutrophils to the dermis in SS is complex and theorized mechanisms are dependent on underlying etiology. Normal neutrophils require TNF-α activated endothelium which leads to neutrophil rolling and attachment via interdependent interactions with selectins, intercellular cell adhesion molecules (ICAM), and integrins (552). These surface linking molecules in concert with inflammatory molecules, including TNF-α and IL-1β, result in normal neutrophil extravasation into tissue. In hematologic malignancy, myeloid blast cells have increased expression of surface adhesion receptors and can induce non-activated endothelial cell adhesion to express receptors leading to accumulation of leukemic cells (553). These cells further promote recruitment, accumulation and tissue invasion by secreting inflammatory cytokines including TNF-α and IL-1β (553). Leukemia cutis, a paraneoplastic tissue invasion of leukemic cells, is well-recognized and has been coexistent in patients with SS and within SS lesions (554–556). Potential mechanisms include dysfunctional malignant cells activating adhesions and creating an inflammatory environment suitable for innocent bystander neutrophils to extravasate, creating SS lesions. Alternatively, cancer therapy, or paraneoplastic stimulatory factors may result in the maturation of leukemia cutis cells into the mature neutrophils within SS lesions. In non-malignant SS associated with other inflammatory conditions, a similar pathologic inflammatory environment could be responsible for localization and infiltration of neutrophils.

### Dysfunctional Immune Mediators

**Figure 1:**
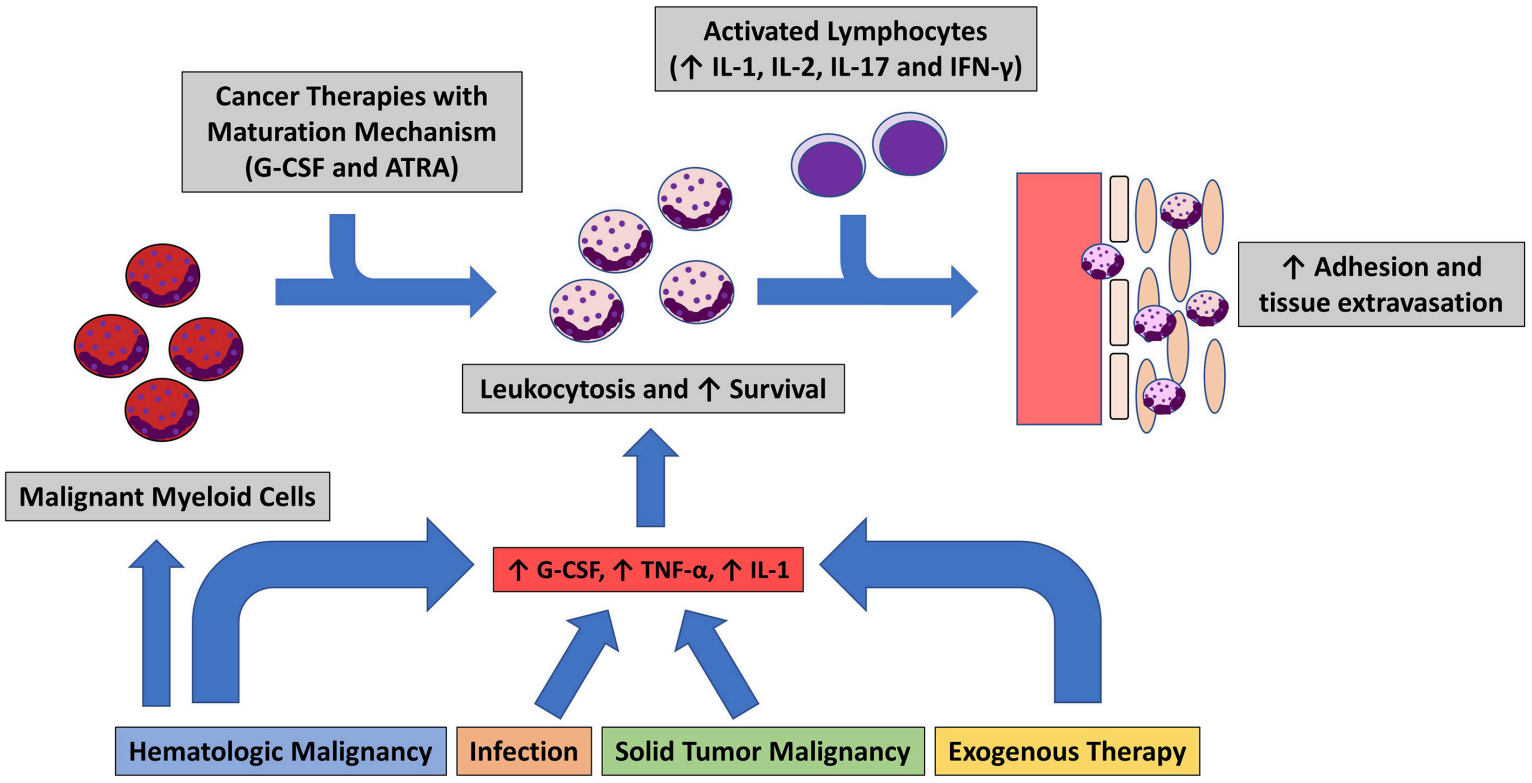
Summary of the hypothesized of Sweet's Syndrome Pathogenesis. Inciting event leads to inflammatory state in which neutrophils mature and proliferate. Lymphocytic cytokine response leads to dermal localization.

The role of a dysfunctional innate immune response in SS is well-established, but evidence is emerging that the adaptive immune system has a significant role. In classic SS, lymphocytes, specifically Type 1 helper T cells (Th1), have been theorized to be responsible for neutrophil activation and localization. This is evidenced by elevated serum levels of Th1 cytokines including IL-1α, IL-1β, IL-2, and IFN-γ (557). Further investigation utilizing immunohistochemical stains has shown a significant presence of these Th1 cytokines and a relative reduction of Type 2 helper T cell (Th2) markers in SS dermal lesions. This suggests hyperexpression of Th1 cells and a comparative suppression of Th2 cells (137, 558, 559). Th1 cells secrete TNF-α and INF-γ, which are potent neutrophil recruiters and activators. Proinflammatory T helper 17 (Th17) cells and related cytokines have also been identified as a pathologic agent in SS (559–562). The role of Th17 cells is most well studied in one of the most prevalent autoinflammatory diseases: psoriasis (563). Th17 produces multiple inflammatory molecules, including interleukin 17 (IL-17). IL-17 works synergistically with TNF α, IL-1β, and IFN-γ to create an inflammatory response and recruits and localizes neutrophils by inducing adhesion molecules, and chemoattractants such as IL-8 (564). Interactions with TNF α and IL-17 induces basement membrane remodeling via pericytes and neutrophils (565). In this SS driven remodeling process, matrix metalloproteinases (MMPs) are significantly upregulated. Upon inhibition of MMP-3, there is a reduction of neutrophil chemotaxis and extracellular matrix degradation (565). The production of G-CSF and GM-CSF are enhanced by IL-17, which leads to activation and proliferation of neutrophils (566, 567). Additional pro-inflammatory markers elevated in SS include: CD40/CD40 ligand, CD56, G-CSF, myeloperoxidase, IL-5, IL-8 IL-12, IL-13, L-selectin, MMP-2, MMP-9, Sialic acid-binding immunoglobulin-type lectin (Siglec) 5, Siglec 9, Transforming growing factor β (TGF-β), TIMP-1, TNF α, and VEGF (127, 524, 558–560, 562, 568, 569). Significant levels of CD56, a Natural killer cell marker, CD40/CD40 ligand, and IFN-γ may indicate the role of antigen presenting cells, as well as a cross-link between the robust innate and adaptive immune response in SS (570). Further evidence of adaptive immunity involvement is suggested by SS remission following treatment with therapies targeting adaptive cell processes including corticosteroids, cyclosporine, IVIG, rituximab, and vedolizumab (121, 132, 571–576). Table 4 summarizes cytokines and inflammatory markers documented in SS. Figure 1 shows the proposed multifactorial mechanism of disease.

**Table 4:** Inflammatory and signaling molecules elevated within lesional dermis and serum.

**Elevated in dermis**	**References**	**Elevated in serum**	**References**
Interleukin-1β	(137, 559)	Interleukin-1α	(557)
Interleukin-4	(558)	Interleukin-1β	(557)
Interleukin-5	(558)	Interleukin-2	(557)
Interleukin-8	(559, 560, 562)	Interleukin-6	(127, 568)
Interleukin-10	(561)	Interferon γ	(557)
Interleukin-12	(558)	G-CSF	(127, 524, 568, 569)
Interleukin-13	(558)	TNF-α	(568)
Interleukin-17	(559, 560, 562)		
Interferon γ	(558)		
MMP-2	(559, 560, 562)		
MMP-9	(560, 562)		
Myeloperoxidase	(560, 562)		
Siglec 5	(559)		
Siglec 9	(559)		
TGF-β	(561)		
TNF-α	(559, 560, 562)		
TIMP-1	(559)		
VEGF	(560, 562)		

*G-CSF, Granulocyte-colony stimulating factor; MMP, Matrix metalloproteinase; Siglec, Sialic acid-binding immunoglobulin-type lectin; TGF-β, Transforming growth factor-β; TNF-α, Tumor necrosis factor-α; VEGF, Vascular endothelial growth factor*.

### Genetic Contributions

There is a growing body of knowledge regarding the genetic contributions in neutrophilic dermatoses including SS. Genetic susceptibility to the SS variant, neutrophilic dermatosis of the dorsal hands, in HLA-B54 positive Japanese individuals has been reported (577). Additional evidence of genetic co-susceptibility and possible mechanisms of SS have been described in synovitis, acne, pustulosis, hyperostosis, osteitis (SAPHO) syndrome, chronic recurrent multifocal osteomyelitis (CRMO), and Majeed syndrome (289, 506, 578, 579). There have been several links between SS and Familial Mediterranean fever (FMF) (425, 580). FMF is an inherited disease in which mutations in the MEFV gene. The MEFV gene is the causative defect identified in FMF, and it is responsible for the expression of pyrin (581). In a non-pathologic state, pyrin, an intracellular pattern recognition receptor, forms the inflammasome complex in response to infections or changes in cellular homeostasis, leading to splicing and secretion of IL-1β (581, 582). Mutations to MEFV as seen in FMF and neutrophilic dermatoses leads to a pathogenic inflammatory response. FMF and SS have coexisted in the same patients and genetic analysis has revealed heterozygous mutations of MEFV in SS (425, 580).

Mutations in isocitrate dehydrogenase 1 (IDH1) have been identified as a possible connection to SS pathogenesis in malignancy (583). IDH1 catalyzes reactions leading to alterations in histones and DNA, causing differential gene expression (584). In myeloproliferative diseases mutations to IDH1 leads to epigenetic chaos as a result of DNA hypermethylation, which leads to abnormal transcription of numerous genes (583). Protein tyrosine phosphatase non-receptor type 6 (PTPN6) plays an essential role in the proliferation and signaling of cells within the immune system (585). Mutations leading to the disruption of normal function of PTPN6 have been identified in hematologic malignancies and neutrophilic dermatoses in mice models (586–590). Alteration of PTPN6 has also been identified in SS patients through DNA sequencing analysis (591). The evidence to date suggests that SS is a polygenic process but dysfunctional activation of the inflammasome and IL-1β pathway offers a unifying mechanism.

### Model of Pathogenesis

The pathogenesis of SS is complex and multifactorial, the different components discussed do not provide a unifying pathway. The most complete model is within the subset of SS patients with hematologic malignancies. The pre-existing myeloid dysfunction and disruption in normal cytokine and stimulating factors provide the environment necessary for aberrant neutrophil activation and inflammation. When patients with hematologic malignancies undergoing treatment develop SS a proposed mechanism is transformation and maturation of dysfunction leukemic cells which continue to exhibit inappropriate activity. In classic SS and drug-induced SS, an inciting stimulus such as an antigen in an individual with a genetical predisposition likely creates a similar pro-inflammatory state resulting in SS. The rarity of SS and the lack of robust experimentation is a major restraint in understanding the disease pathogenesis.

## Treatment Approaches

Management of SS is partially reliant on the underlying association, but given the severe presentation and possibility of non-modifiable etiology, prompt treatment is usually warranted (592). In drug induced SS, identification and removal of the offending agent is beneficial but does not negate the need for treatment. First line treatments for SS include corticosteroids and other agents such as potassium iodide or colchicine. Second line agents for SS include indomethacin, clofazimine, cyclosporin, and dapsone (592, 593). The effectiveness of these medications with differential mechanisms of action highlights the role of both adaptive and innate cells in the pathogenesis of SS (594–596). With advances in our understanding of the pathophysiology of neutrophilic dermatoses, especially the role of TNF-α and IL-1β, the use of targeted therapy with IL-1 and TNF-α inhibitors has been effective (323, 593, 597–603). There have been reports of several novel treatments for SS, including granulocyte and monocyte adsorption apheresis, but due to the rarity of SS and the effectiveness of established treatments there have been limited investigations into these alternative treatments (604).

## Conclusions and Future Directions of Research

Over the last half century, SS has retained its defining characteristics while medical advances and scientific discovery have led to a better understanding of disease mechanisms and associations. The clinical similarity of SS with other neutrophilic driven autoinflammatory entities is challenging in clinical grounds as the diagnostic criteria is not applicable in atypical presentations or overlapping autoinflammatory dermatoses. Relations with medications, inflammatory diseases, and malignancy have been established and expanded on. Dermal neutrophil clonality and transformation of malignant myeloid progenitors into infiltrating neutrophils provides evidence for an etiology in myeloproliferative disease and offers insight into future directions of research. Investigations into immunologic signaling pathways have improved our understanding of the interrelationships between inflammation and disease pathogenesis. The involvement of IL-17, IL-1β, and inflammasome activation are of great interest in neutrophilic dermatoses including the utilization of targeted therapies. As this pathway is ubiquitous throughout inflammatory processes, an emphasis on better understanding its mechanism will be paramount to advances in not only SS but throughout medicine. As genetic analysis and gene profiling techniques are revolutionized and optimized, new discoveries on the role of genetic susceptibility, heritability, and more specific markers of neutrophilic dermatoses will be on the horizon

## Author Contributions

MH and AO-L conceived the idea for this work and performed the literature review on the subject. MH compiled the data with AO-L oversight. MH and AO-L wrote the manuscript and finalized the published version.

### Conflict of Interest Statement

The authors declare that the research was conducted in the absence of any commercial or financial relationships that could be construed as a potential conflict of interest.
